# High-Output Heart Failure Contributing to Recurrent Epistaxis Kiesselbach Area Syndrome in a Patient With Hereditary Hemorrhagic Telangiectasia

**DOI:** 10.1177/2324709617692833

**Published:** 2017-02-01

**Authors:** Venugopal Brijmohan Bhattad, Jennifer N. Bowman, Hemang B. Panchal, Timir K. Paul

**Affiliations:** 1 East Tennessee State University, Johnson City, TN, USA

**Keywords:** high-output heart failure, epistaxis, hereditary hemorrhagic telangiectasia, recurrent epistaxis Kiesselbach area syndrome, anemia

## Abstract

Hereditary hemorrhagic telangiectasia (HHT), also known as Osler-Weber-Rendu syndrome, is a rare genetic blood disorder that leads to abnormal bleeding due to absent capillaries and multiple abnormal blood vessels known as arteriovenous malformations. A feature of HHT is high-output heart failure due to multiple arteriovenous malformations. High-output heart failure can lead to recurrent epistaxis Kiesselbach area syndrome (REKAS), further exacerbating heart failure through increased blood loss and resultant anemia. We report a patient with HHT who presented with high-output heart failure contributing to REKAS. In patients with REKAS, we propose if anemia is present, REKAS can be avoided by correcting the anemia by increasing the hemoglobin level to greater than 9 to 10 g/dL. This decreases hyperdynamic circulation and reduces pressure in the blood vessels of the nose.

## Introduction

Hereditary hemorrhagic telangiectasia (HHT) is a rare autosomal dominant blood disorder that is characterized by multiple telangiectasia (dilated blood vessels in mucous membranes) and arteriovenous malformations (AVMs), which are direct connections between arterioles and venules. The prevalence is approximately 1 in 5000 to 10 000.^1^ HHT most commonly manifests as recurrent epistaxis, but it can also manifest as bleeding in the gastrointestinal, pulmonary, or cerebral circulations.^2,3^ The typical presentation is epistaxis during puberty, with cutaneous telangiectasia occurring later in life, usually by the fourth decade.^4^ The molecular basis for HHT is a mutation in the endoglin (ENG) gene or activin receptor-like kinase-1 (ALK1) gene involved in angiogenesis.^5^ Endoglin is a coreceptor and ALK1 is a receptor for the TGF-beta family.^6^ A mutation in MADH4, a gene which codes for Smad4, has also been linked to HHT.^7^ These 3 genes are part of the TGF-beta superfamily signaling pathway. Endoglin and ALK1 are found in the vascular endothelium and help maintain vascular integrity; mutations in these genes lead to vascular dysfunction and the multiple telangiectasia found in HHT.^7,8^

The diagnostic criteria used to diagnose HHT, known as the Curaçao criteria, are listed in Table 1. A definitive diagnosis must include at least 3 of the 4 criteria.^9,10^

**Table 1.** Curaçao Criteria for Diagnosis of HHT^9,10^.

1. Spontaneous recurrent epistaxis
2. Multiple telangiectasia in typical locations (eg, oral mucosa, palms, nose)
3. Proven visceral AVMs in places such as the gastrointestinal, pulmonary, or cerebral circulations
4. First-degree family member with HHT

Abbreviations: HHT, hereditary hemorrhagic telangiectasia; AVM, arteriovenous malformation.

Large vessel involvement, such as pulmonary AVMs, can occur in 15% to 20% of patients and can lead to high-output heart failure. The mechanism of action involves the prolonged increase in blood flow from the AVMs, which leads to changes in the elasticity of the blood vessel walls, leading to vasodilation and lower systemic vascular resistance. This ultimately ends with high-output heart failure.^11^

High-output heart failure is defined by the parameters found in Table 2. Etiologies of high-output heart failure include systemic arteriovenous fistulas, hyperthyroidism, anemia, beriberi, dermatologic disorders (eg, psoriasis), renal disease, hepatic disease, skeletal disorders (eg, Paget disease, multiple myeloma), and sepsis.^12-14^

**Table 2.** High-Output Heart Failure Parameters^12^.

1. Cardiac output >8 L/min or cardiac index >3.9 L/min/m2
2. Clinical signs and symptoms of heart failure
3. Echocardiogram with preserved ejection fraction of >50%
4. Mixed venous oxygen saturation of >75%

Recurrent epistaxis Kiesselbach area syndrome (REKAS), first described in the literature in 1985, refers to the area in the nasal septum where the anterior and posterior ethmoidal artery, sphenopalatine artery, greater palatine artery, and septal branch of the superior labial artery join to form a vascular plexus.^15,16^ Since the area is so vascularized, patients with HHT are prone to recurrent epistaxis in the region. Blood vessels appear to be dilated in the Kiesselbach plexus, and patients may have an anterior septal deformity.^17^

## Case Presentation

A 73-year-old female with a known history of HHT and multiple blood transfusions presented to emergency room with recurrent and recent worsening of epistaxis. Over the past week, she had increased shortness of breath with exertion, and black tarry stools. She denied any chest pain, syncopal episodes, abdominal pain, nausea, vomiting, or hematemesis. She had a past medical history significant for iron-deficiency anemia and a past surgical history significant for endoscopic nasal exam with cauterization. She denies tobacco, alcohol, or illicit drug use. Her family history was negative for blood disorders. Her home medications included amlodipine, cyanocobalamin, conjugated estrogen, levothyroxine, and venlafaxine.

The patient was afebrile with a heart rate of 98/min, blood pressure of 117/72 mm Hg, and respiratory rate of 18/min. Overall, she appeared weak and pale. Her physical examination demonstrated nares with dried blood; palate, tongue, and lip telangiectasia; multiple skin telangiectasia; elevated jugular venous pressure up to angle of jaw; grade III/VI systolic ejection murmur; and bounding peripheral pulses. She also had bilateral basilar crackles posteriorly and 2+ pedal edema.

Complete blood count showed hemoglobin of 7.1 g/dL and hematocrit of 24%, with an otherwise normal white blood cell count and platelet count. Mean corpuscular volume and basic metabolic panel were unremarkable. Endoscopy done 3 years prior to this admission and a computed tomography (CT) of chest done 2 years prior were consistent with multiple AVMs in the gastrointestinal tract and lungs, respectively. A CT of the sinuses of the head done 4 years prior showed mucosal thickening of the left maxillary and sphenoid sinus. A previous stress echocardiogram done 3 years prior for an episode of chest pain was negative for ischemia with an ejection fraction of 72%.

During the course of the patient’s hospital stay, she received a total of 4 units of packed red blood cell transfusions with resolution of her epistaxis with improved hemoglobin from 7.1 g/dL on admission to 9 g/dL on discharge.

## Discussion

Our patient had fulminant signs, and symptoms, of high-output heart failure with worsening and recurrent epistaxis in the setting of AVMs and acute anemia. She received multiple units of packed red blood cells, and hematology recommended keeping patient’s hemoglobin at 9 to 10 g/dL. This eventually resolved the patient’s epistaxis. However, whenever the patient had a decline in hemoglobin to less than 9 g/dL, she would begin to experience dyspnea and fatigue, and she would present with recurrent epistaxis.

The basic pathophysiology of high-output heart failure is an issue with decreased systemic vascular resistance, either through vasodilation of the systemic vessels or arteriovenous shunting.^12^ This leads to decreased blood pressure, which activates the sympathetic nervous system. Via this mechanism, the heart compensates for the decreased blood pressure by increasing heart rate and thus cardiac output. Additionally, the renin-angiotensin-aldosterone (RAA) pathway is activated, leading to water and sodium retention.^18^ Therefore, the ejection fraction is still preserved in high-output heart failure; however, the body is unable to deliver enough oxygen to the tissues, leading to heart failure despite the hyperdynamic circulation.

**Figure 1.**
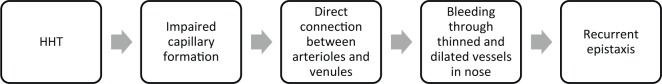
Proposed mechanism of action causing recurrent epistaxis in HHT.

Even though many peripheral parts of the body receive decreased blood flow due to systemic vasodilation, the area of Kiesselbach’s plexus receives extra pressure due to the increased vascularity of the area and the close proximity to the pumping force of the heart. Combining the increased pressure with the thinner and more delicate blood vessels in the nasal septum leads to an increased risk of recurrent epistaxis^2^ (Figure 1).

**Figure 2.**
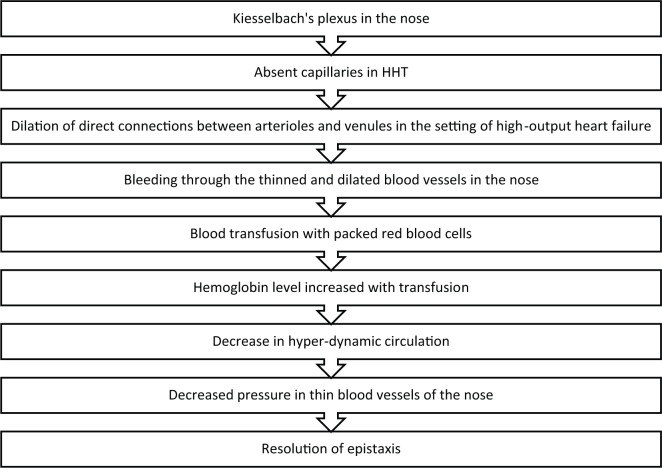
Proposed mechanism of avoiding REKAS in HIT by correcting anemia.

By keeping the patient’s hemoglobin greater than 9 g/dL would decrease the hyperdynamic circulation by decreasing the compensatory mechanisms of the heart and decreasing activation of the RAA pathway. This exerts less pressure on the thinned and dilated blood vessels of Kiesselbach’s plexus, leading to resolution of the epistaxis (Figure 2). This was a relatively easy therapy for this patient, and she was able to be discharged 2 days after admission with complete resolution of symptoms.

The causes of high-output heart failure in our patient include irreversible causes, such as AVMs, and reversible causes, such as iron-deficiency anemia secondary to blood loss. Many causes are described in the literature and are outlined in Tables 3 and 4. Specifically, our patient had CT evidence of multiple AVMs. The prolonged increase in blood flow caused changes in the elasticity of the blood vessel walls, which led to vasodilation and lower systemic vascular resistance and thus triggering the high-output heart failure pathway. The reversible cause of anemia is related to the physiological changes of the body in response to decreased oxygenation and tissue perfusion, once again triggering a compensatory increase in cardiac output and activation of the RAA pathway. In our patient, packed red blood cells transfusion increased oxygenation and tissue perfusion, and lessening the patient’s high-output heart failure.

**Table 3.** Reversible Causes of High-Output Heart Failure and Mechanism of Action^12-15,18-21^.

Reversible Causes of High-Output Heart Failure	Mechanism
Anemia	Physiological changes to maintain oxygenation and tissue perfusion, such as increase in cardiac output and activation of the renin-angiotensin-aldosterone pathway
Sepsis	Systemic vasodilation and increased cardiac output
Hypercapnea (eg, COPD, connective tissue disorders, interstitial lung disease)	Reduction in systemic vascular resistance
Hyperthyroidism	Hyperdynamic circulation via tachycardia and increased cardiac output
Beriberi (thiamine deficiency)	Vasodilation
Pregnancy	Increased cardiac output and blood volume, placenta acts as arteriovenous shunt, vasodilation
Obesity	Increased metabolic activity from excess adipose, causing increase in cardiac output and blood volume; also element of hypercapnea
Hepatic disease (eg, cirrhosis)^a^	Splanchnic vasodilation, decreased systemic vascular resistance
Carcinoid syndrome^a^	Vasodilation
Myeloproliferative disorders^a^	Increased metabolic demand for tissue perfusion via changes in tissue metabolism
Dermatological diseases (eg, psoriasis)	Increased blood flow to the skin due to cutaneous dilatation

Abbreviation: COPD, chronic obstructive pulmonary disease.

a May be reversible or irreversible, depending on underlying cause, severity, and treatment prognosis.

**Table 4.** Irreversible Causes of High-Output Heart Failure and Mechanism of Action^12-15,22-24^.

Irreversible Causes of High-Output Heart Failure	Mechanism
Systemic arteriovenous fistulas or AVMs	Prolonged increase in blood flow causes changes in elasticity of the blood vessel wall, leading to vasodilation and lower systemic vascular resistance
Paget’s disease	Associated with AVMs, increased metabolic activity of affected bone leading to increased blood flow to area
Multiple myeloma	Associated with AVMs
McCune Albright syndrome	Associated with AVMs

Abbreviation: AVM, arteriovenous malformation.

Iron-deficiency anemia due to recurrent blood loss (reversible), and AVMs (irreversible), can cause hyperdynamic circulation and high-output heart failure, which, over a period of time, can trigger REKAS due to dilatation of the Kiesselbach venous plexus in HHT.^12^ To date, no standard of treatment has been described in the literature. Surgical treatments such as suturing of Little’s area or liver transplantation in HHT are considered more aggressive and are associated with surgical risks.^25-27^ Radiofrequency ablation of Kiesselbach’s plexus can lead to septal perforation and is not a definitive treatment.^28^ HHT is associated with increased levels of vascular endothelial growth factor (VEGF) leading to the development of multiple telangiectasia and AVMs.^29^ Bevacizumab, a VEGF inhibitor, is a most expensive drug and not widely studied to control epistaxis in HHT, due to rareness of this disease. Studies have found it to be safe to use in HHT, but it is not currently approved by the Food and Drug Administration in the treatment of epistaxis in HHT until more studies are suggestive of actual benefit of a decrease in epistaxis.^30-33^ Thalidomide, a platelet derived growth factor B stimulator, is another drug proposed to treat epistaxis and gastrointestinal bleeding in HHT.^34,35^ However, thalidomide has a significant side effect profile of increased fatigue and neuropathy, and both bevacizumab and thalidomide’s efficacy are not established with Phase 2 clinical trials.^29-34^ Hence, we propose that REKAS in HHT can be avoided with correction of the reversible causes of high-output heart failure by correcting the anemia through increasing hemoglobin level to approximately greater than 9 to 10 g/dL.

## Conclusion

High-output heart failure can lead to REKAS, further exacerbating heart failure through increased blood loss and resultant anemia. To date no standard treatment has been proposed. If anemia is present, REKAS can be avoided by correcting the anemia with a target hemoglobin level of nearly 9 to 10 g/dL or greater.
